# Access to health for refugees in Greece: lessons in inequalities

**DOI:** 10.1186/s12939-016-0409-6

**Published:** 2016-08-02

**Authors:** Antonis A. Kousoulis, Myrsini Ioakeim-Ioannidou, Konstantinos P. Economopoulos

**Affiliations:** 1 Society of Junior Doctors, Athens, Greece; 2 Faculty of Epidemiology and Population Health, London School of Hygiene & Tropical Medicine, 10 Norwood Avenue, London, HA0 1LY UK; 3 Medical School, University of Athens, Athens, Greece; 4 Massachusetts General Hospital, Harvard Medical School, Boston, MA USA

**Keywords:** Access, Conflict, Greece, Inequality, Migrants, Refugee, Syria

## Abstract

Eastern Greek islands have been direct passageways of (mainly Syrian) refugees to the European continent over the past year. However, basic medical care has been insufficient. Despite calls for reform, the Greek healthcare system has for many years been costly and dysfunctional, lacking universal equity of access. Thus, mainly volunteers look after the refugee camps in the Greek islands under adverse conditions. Communicable diseases, trauma related injuries and mental health problems are the most common issues facing the refugees. The rapid changes in the epidemiology of multiple conditions that are seen in countries with high immigration rates, like Greece, demand pragmatic solutions. Best available knowledge should be used in delivering health interventions. So far, Greece is failed by international aid, and cross-border policies have not effectively tackled underlying reasons for ill-health in this context, like poverty, conflict and equity of access.

## Background

It was Christmas Eve when 27-year-old S., musician, and his partner O., singer, entered our clinic for a quick check. O. was 5 months pregnant, and they had just made it across the Mediterranean from the coast of Turkey to Lesbos. They admitted that they were feeling that this dangerous path was their only option. Holding a small yellow plastic box in her hands, healthy O. explained that this box was their whole life: a couple of cassettes with their music recordings. With this in hand they would pursue their dream in Germany. If they make it there that is.

It is almost impossible to be confident about the future when going through this astonishing journey that many Syrian refugees have to these days. They are pursuing their “*right to seek and to enjoy in other countries asylum from persecution*”. Providing a safe passageway to refugees is vital, but at the same time this is merely enough, as the offer of basic medical care is insufficient in most of the host intermediary countries [1].

### Accessing health care

Greece is no exception. As a country it has consistently relied on a healthcare system following much costly processes. Lack of equity of access to health care has been a fundamental problem of the system, and primary care services, despite long available, have not been redesigned into an integrated model [2]. These have been aggravated by the economic crisis [3]. It has been suggested that interventions should be identified that improve performance, production, efficiency and equity. Lack of these leads to key questions that remain unanswered [2]. Should Greece follow the “exclusive” strategy of implementing interventions taking into account differences in biology, life course, language culture etc., or the “inclusive” approach of adapting the existing routine health and preventive services [4]? At the moment, it remains unable to provide either.

### Organizing the refugees’ stay

Leaving the rest of his family behind in “safe” Damascus, perfume maker C., with his wife and 6-month-old daughter, was now optimistically heading to the Netherlands. They left Latakia on foot, hitchhiked in Turkey and ended up with no luggage in Lesbos. He was very vocally grateful for the medical support he received from the volunteers of the Greek island.

Dozens of Non-Governmental-Organizations (NGOs) have operated throughout the year on the island of Lesbos. Even during the 2015 Christmas holidays there was an unabated influx of 4,000 refugees arriving per day, mainly in the late-night hours.^1^ Along the coast, tens of volunteers stood by to support offering thermal blankets, water and biscuits. They have set up a simple mobile-based communication network, despite minimal coordination between NGOs.

Volunteers ensured that refugees can be accommodated in the camp. Families with young children, pregnant women, and current patients received priority treatment. Almost 6,000 were hosted in the *hotspot* at any one time, in facilities with capacity of 850 which unavoidably remained unheated and unsanitary. The rest spent their nights in small huts provided for by UNHCR or outside, under the olive trees of the surrounding area. Volunteers and local residents also distributed limited quantities of food, tea and water. Lack of dry clothing or blankets has been a known issue with refugees complaining for the lack of hygiene, food or provisions for their necessary religious practices. Adults despair quietly, while children cry inconsolably, deprived of any proper sleep.

### Healthcare provisions

Refugee health in Lesvos is not dissimilar to what can be expected [5]. On arrival, trauma and hypothermia are the main emergencies. Anyone unwell or injured is transferred to a primary care medical tent for basic physical examination. Volunteer physicians and nurses from across the world offer their services in Lesvos. Some NGOs (e.g. Médecins Sans Frontières, Doctors of the World) have created clinics within the camps. Medical services are offered under adverse conditions.

Based on the handwritten notes kept in a 24/7 clinic ran by Off Track Health charity, refugees’ most common symptoms are fever, chills, sore throat, diarrhea, chest pain, abdominal pain. Many refugees suffer from infections, bronchiolitis, asthma attacks, or trauma-related injuries. Some young mothers are too stressed to breastfeed their newborns and are assisted by volunteers of “Save the Children” NGO. Any medical support provided by medical personnel in Moria, though basic and inadequate, is treated with profound gratitude by the refugees.

War experiences and exile-related stressors cause severe mental health problems to many refugees, including anxiety and depression [6]. Communication difficulties often make it impossible for volunteers to provide emotional support. Tens of thousands of refugees will remain trapped in – the unable to offer decent options to asylum seekers – Greece, homeless and unemployed.

## Conclusion

Implementing evidence based interventions to provide access to screening and prevention is the gold standard in public health care. However, the rapid changes in the epidemiology of multiple conditions that are seen in countries with high immigration rates, like Greece, demand more pragmatic solutions. For example, following a simple decision-making tool at point of care could increase participation in screening in communities known to have disproportionately high populations of immigrants or refugees who may have a language barrier [7]. Refugees can be a target audience for which just the best available knowledge can be used. This would require, however, coordinated international expertise and support for Greece. So far, EU foreign and economic policies have failed to effectively tackle underlying reasons for ill-health: i.e. poverty, conflict, equity of access [1].

Fifteen-year-old K. was using crutches when he entered our clinic, alongside his family. He had difficulty to walk and asked for his leg sutures to be removed; a typical victim of the ongoing war in Syria. Until systematic solutions can be provided, we will only be able to offer hope to 5-year-old N., K.’s little sister, who, while being examined in the primary care tent, took a stethoscope in her hands and said she would become a doctor “when all this is over”.

## Abbreviations

NGO, Non Governmental Organization; UNHCR, United Nations High Commissioner for Refugees

## Footnotes

1 Personal observations and reports from local news.

## References

[1] Razum O, Kaasch A, Bozorgmehr K. From the primacy of safe passage for refugees to a global social policy. Int J Public Health. 2016;4 [Epub ahead of print]. 10.1007/s00038-016-0817-9 27040939

[2] Kousoulis AA, Angelopoulou KE, Lionis C (2013). Exploring health care reform in a changing Europe: lessons from Greece. Eur J Gen Pract.

[3] Karanikolos M, Kentikelenis A (2016). Health inequalities after austerity in Greece. Int J Equity Health.

[4] Razum O, Spallek J (2014). Addressing health-related interventions to immigrants: migrant-specific or diversity-sensitive?. Int J Public Health.

[5] Abu Sa’Da C, Serafini M (2013). Humanitarian and medical challenges of assisting new refugees in Lebanon and Iraq. FMR.

[6] Miller KE, Weine SM, Ramic A (2002). The relative contribution of war experiences and exile-related stressors to levels of psychological distress among Bosnian refugees. Journal of Traum Stress.

[7] Jones M, Ross B, Cloth A, Heller L (2015). Interventions to reach underscreened populations: a narrative review for planning cancer screening initiatives. Int J Public Health.

